# Coactivation of the Pelvic Floor and Gluteus Medius Muscles While Walking and Running in Female Runners

**DOI:** 10.3390/s24051356

**Published:** 2024-02-20

**Authors:** Avelaine Porrón-Irigaray, Elena Sonsoles Rodríguez-López, María Barbaño Acevedo-Gómez, Cristina Ojedo-Martín, María Benito-de-Pedro

**Affiliations:** 1 Department of Physiotherapy, Faculty of Health Sciences-HM Hospitals, University Camilo José Cela, 28014 Madrid, Spain; avelaine@hotmail.com (A.P.-I.); acevedogomez.maria@gmail.com (M.B.A.-G.); mbenito@ucjc.edu (M.B.-d.-P.); 2 Instituto de Biomedicina de Sevilla (IBiS), Department of Physiotherapy, Universidad de Sevilla, 41013 Seville, Spain

**Keywords:** pelvic floor, walking, running, EMG, gluteus, sportswomen

## Abstract

(1) Background: Pelvic-floor-muscle (PFM) activation acts synergistically with multiple muscles while performing functional actions in humans. The purpose of this study was to characterize the activity of the PFMs and gluteus medius (GM) while walking and running in physically active nulliparous females. (2) Methods: The peak and average amplitude of maximal voluntary contractions (MVCs) during 60 s of walking (5 and 7 km/h) and running (9 and 11 km/h) were measured with electromyography of the GM and PFMs in 10 healthy female runners. (3) Results: The activation of both muscles increased (*p* < 0.001) while walking and running. The MVC of the GM was reached when walking and tripled when running, while the PFMs were activated at half their MVC when running. The global ratio of the GM (75.3%) was predominant over that of the PFMs (24.6%) while static and walking. The ratio reached 9/1 (GM/PFM) while running. (4) Conclusion: The GM and PFMs were active while walking and running. The GM’s MVC tripled at high speeds, while the PFMs reached only half of their maximum contraction.

## 1. Introduction

The activation of the pelvic floor muscles (PFMs) is linked to the activity of other muscle groups during certain functional actions, including walking, contributing to continence, and pelvic stability [1]. Therapists commonly prescribe hip abductor exercises, involving the gluteus medius, to improve strength due to their role in maintaining a level pelvis and preventing hip adduction and femoral internal rotation in single-limb support [2,3]. The synergistic mechanism of the PFMs continues to be a subject of study given its complexity, a lack of knowledge, and its importance in containing the increase in intra-abdominal pressure (IAP) in multiple actions [4]. During dynamic movements, the abdominal, paravertebral, or pelvic floor muscles are activated, which increases the IAP [4,5]. If there is PFM competence, a prior contraction occurs to avoid visceral descent in the event of a high IAP [1,4]. In the case of muscle dysfunction, there is a probability of suffering from urinary incontinence (UI) [6,7].

Surface electromyography (EMG) allows us to precisely determine the intensity and frequency of muscle activation during the different phases of walking [8,9]. Since groups of muscle fibers can be selectively activated during a motor gesture, EMG generates specific spectra associated with the conduction speed of the action potential of the motor unit [10]. EMG has allowed us to discover synergies of the PFMs with the abdominal muscles [11], hip abductor muscles [5], and shoulder flexor/extensor muscles [12,13].

The neural connections responsible for these synergies are relevant for the treatment and prevention of UI [14,15], chronic pelvic pain [16,17], readaptation in high-performance sports [18,19,20], or running cycle effectiveness [2,20]. Multiple studies link the synergy of the gluteus maximus muscle (GMM) with the PFMs [5,21,22]. However, there is no evidence regarding the synergy of the gluteus medius (GM) and recording PFM activity. Therefore, we hypothesized that the gluteus medius and pelvic floor are activated while walking and running in female runners but at different intensities. Therefore, the main objective was to compare the activation of the PFMs and GM while walking and running in nulliparous, physically active female runners throughout the gait cycle at different speeds. As a specific objective, it was proposed to determine the peak and average amplitude and muscle ratios of the GM and PFMs in static tests and at different speeds (5, 7, 9, and 11 km/h) in female runners.

## 2. Materials and Methods

### 2.1. Study Design

An analytical observational study was carried out by performing various EMG tests within the same session. The activity of the PFMs and GM was recorded while static and while walking and running at four different speeds. This study was approved by the Ethics Committee of University Camilo José Cela (code 18_FRIU) (Madrid, Spain). All participants received detailed information about this study, including the potential risks and benefits. They provided oral and written informed consent before commencing.

### 2.2. Participants

The participants were female recreational runners (with more than 5 years of experience, consisting of over 20 km/week), were physically active, were nulliparous, were aged 18 to 42 years, had a BMI between 18 and 30 kg/m^2^, were able to read and understand Spanish, and could run on and had experience with running on a treadmill. The exclusion criteria were pregnant women, active or recurrent infection of the genitourinary tract, suffering from a phobia or impossibility of penetration or vaginal hypertonia for which it is not possible to introduce a vaginal probe [5,21,22], experiencing allergies to heavy metals, suffering from an injury or dysmetria of the lower limbs of greater than 0.7 cm [23], and self-reported history of diabetes mellitus, urogynecological or pelvic surgery, pelvic irradiation, pelvic floor dysfunction (e.g., urinary or anal incontinence, or pelvic organ prolapse), hypermobility syndrome or mobility impairments that could affect PFM morphology, stiffness, or contractility. All participants attended a single session for the data collection.

### 2.3. Instrumentation and Data Collection

#### 2.3.1. Sociodemographic, Medical, and Sports Practice Data

The women completed an online questionnaire, where anthropometric information (including age, weight, height, and BMI), medical histories (including common diseases, urinary infection, and gynecological and obstetric history), and sports practice were collected via the International Physical Activity Questionnaire (IPAQ) [24].

#### 2.3.2. Pelvic-Floor-Muscle Assessment: Modified Oxford Scale

Each participant with an empty bladder was placed in the supine position on a stretcher with a pillow under her head, the hips and knees gently flexed (supported with a roller under the knees) and the lumbar spine in a neutral position. A bi-digital examination was carried out with a lubricant, the global voluntary contractibility of the pelvic floor muscles was assessed, and the corresponding score was awarded (from a minimum score of Grade 0, or none, to Grade 5, or strong) [25,26]. If the patient presented an incorrect contraction, they were instructed to perform it correctly, without parasitic contractions or pelvic movement.

#### 2.3.3. Electromyography

The muscle activation of the PFMs [27,28] and GM [29,30] was measured using EMG mDurance^®^ (Granada, Spain), a scientifically validated tool in the field of physiotherapy and sports rehabilitation that combines portable surface electromyography, an operating system, and cloud analysis [31,32]. We aimed to verify the muscular synergy of the two muscles through the autonomous processing and filtering of the EMG signals of muscle activity in real time.

Participants were instructed by an expert physiotherapist at the beginning of each EMG measurement [33,34]. A brief anatomical reminder was offered, and a briefing of the procedures and verbal instructions for the voluntary contraction of the GM and PFMs were given [35]. Audios were recorded to request the appropriate muscle activation with a tone, rhythm, and duration according to the test to provoke the same stimulus [36].

The electrodes of the first mDurance^®^ device were connected using two independent channels to each GM to record the EMG activity [29,37,38] according to the recommendations of the SENIAM (surface-electromyography non-invasive assessment method) protocol [39]. The second device was used to record the muscle-activity EMGs of the PFMs [28,35]. A Perisize 4^®^ single-use vaginal probe (Neen HealthCare, Dereham, United Kingdom) with four electrodes, two independent tracks, two channels, and a total weight of 16 g was employed for this purpose. The probe was introduced vaginally by a specialist physiotherapist, the same researcher who ensured the correct contraction of the PFMs. Likewise, the recommendations of the SENIAM [39] were followed.

For all EMG data, the sampling frequency was 2 kHz with an amplification gain of 1000, and the signals from the two devices were wirelessly sent to a computer (via Bluetooth) using two sensors to record and evaluate the EMGs and range of movement. The raw EMG data were band-pass-filtered between 20 and 450 Hz using a fourth-order Butterworth filter [35,38].

**Figure 1:**
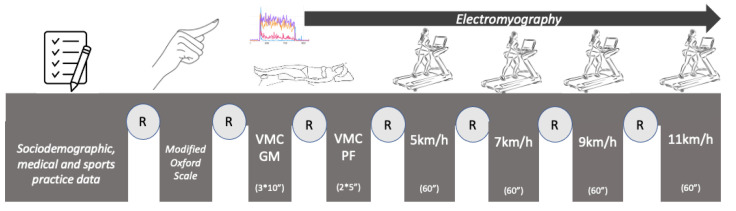
Data collection. R, rest; MVC GM, maximum voluntary isometric contraction of the GM; 3 × 10”, 3 contractions for 10 s; MVC PFM, maximum voluntary isometric contraction of the PFM; 2 × 5”, 2 contractions for 5 s.

The following measurements were taken after a 10 min rest period in the supine position (Figure 1):

- Maximum voluntary isometric contractions (MVCs) of the GM [40]: Three MVCs were measured, each held for 10 s and with a 60 s rest between each contraction [41,42]. From the data reported in the EMG, the maximum amplitude (MVCpeak) and the result of the arithmetic mean of the three maximum peaks of each contraction for the right and left GM muscles (MVCpeak_GM_RGM and MVCpeak_GM_LGM) and for the right and left PFMs (MVCpeak_GM_RPFM and MVCpeak_GM_LPFM) were calculated. The mean amplitude (MVCmean) was the result of the average muscle activity during the 10 s of each contraction for the right and left GM muscles (MVC-mean_GM_LGM and MVCmean_GM_RGM) and for the right and left PFMs (MVCmean_GM_RPFM and MVCmean_GM_LPFM).
- Maximum voluntary contractions (MVCs) of the PFMs [35,43,44]: Two MVCs were measured, each held for 5 s and with a 30 s rest between each contraction [44]. The reference value set as 100% for EMG normalization (MVCpeak) was calculated as the mean of the peak values of two maximum voluntary contractions (30 s) for the GM (MVCpeak_PFM_RGM and MVCpeak_PFM_LGM) and the PFMs (MVCpeak_PFM_RPFM and MVCpeak_PFM_LPFM). The mean amplitude (MVCmean) was the result of the average muscle activity during the 30 s of each contraction for the right and left GM muscles (MVCmean_PFM_RGM and MVCmean_PFM_LGM) and for the right and left PFMs (MVCmean_PFM_RPFM and MVCmean_PFM_LPFM).
- Muscle ratio: The muscle relationships were calculated by dividing the muscles into pairs. For example, the ratio of the average amplitude during the maximum-GM-contraction test of the right PFM to the right GM was calculated as “Ratio_RGM_RPFM = MVCmean_GM_RGM/MVC-mean_GM_RPFM”. This was replicated for each value.

#### 2.3.4. Recording of EMG Activity at Different Walking Speeds

To perform the dynamic assessment, the women dressed in comfortable sports clothing and regular shoes and wore loose belts to hold the two mDurance^®^ sensors. A treadmill (Technogym^®^, Excite Live Run model, Cesena, Italy) was used to assess gait and allowed us to regulate the speeds and intervals necessary to record the gait at four different speeds. Throughout the test, the participants were instructed not to activate the PFM voluntarily, normalize their breathing, and not to speak while executing the tasks [35,37,45].

The participants started walking at 5 km/h to familiarize themselves with the treadmill for 60 s. We then proceeded to record the participants while they walked at 5 and 7 km/h for 60 s and ran at 9 km/h and 11 km/h with two EMG sensors (PFM and GM). A total pause of 60 s between the different speeds was established. The data acquisition began after resting for 60 s and as soon as they reached the respective speed until the 4 recordings at the different speeds were completed [35,37,45].

From the data reported in the EMG, the maximum amplitude (peak) was calculated as the mean of the three maximum peaks of each test for the GM (5Peak_GM, 7Peak_GM, 9Peak_GM, and 11Peak_GM) and the pelvic floor muscles (5Peak_PFM, 7Peak_PFM, 9Peak_PFM, and 11Peak_PFM). The mean amplitude (mean) was the result of the average muscle activity during the 60 s of each test for the GM (5Mean_GM, 7Mean_GM, 9Mean_GM, and 11Mean_GM) and the pelvic floor muscles (5Mean_PFM, 7Mean_PFM, 9Mean_PFM, and 11Mean_PFM). Values were obtained for each muscle in isolation; therefore, the values corresponding to the right and left GM muscles and right and left PFMs are reported.

The data were expressed as percentages with respect to the MVCs of the GM and PFMs [38,46]. The results of the maximum and average amplitudes of each rhythm were compared with the amplitude reached during the MVC of each muscle. For example, the percentage of the maximum amplitude in the activation of the right GM at a 5 km/h pace with respect to the maximum amplitude of the MVC in the supine test was calculated using the following equation: “%Peak_RGM_5 km/h = (5Peak_RGM × 100)/MVCpeak_GM_RGM”. This was replicated for each value.

The muscle ratios were calculated via the pairwise division of the muscles. For example, the ratio of the average amplitude of the right pelvic floor to the right GM during the 5 km/h test was calculated as follows: “Ratio_5 km_RGM_RPF = 5Peak_RGM/5Peak_RPF”. This was replicated for each value.

### 2.4. Statistical Analysis

The sample size was calculated using the G*power 3.1 software (Kiel University, Kiel, Germany). Based on previous research [4,5,13,35] in the same population, a two-tailed hypothesis with an effect size of 0.80, α-error probability = 0.05, and statistical power = 0.90 was employed for the sample size calculation. According to these parameters, ten participants were necessary to complete this study.

Data were analyzed using the IBM Statistics Package for Social Science, v.26 (IBM Corp, NY, USA). Data are reported as means (standard deviation) with a 95% confidence interval (95% CI) or as percentages. Before performing the analysis, Shapiro–Wilk tests were used to check the normality of the variables; they all met the normality criteria (*p* > 0.05). Due to the small sample size, the Friedman test was used to analyze the different measurement times of each muscle in each test. The Wilcoxon test was used when a pairwise comparison of the time between static and different speeds was necessary. Possible associations between study measures were tested with Pearson’s product-moment correlation coefficient analysis or the Spearman rank test. A *p*-value of <0.05 was considered statistically significant.

## 3. Results

Twelve female runners were recruited, two of which were excluded from the analysis, one due to vaginismus and an inability to insert the intracavitary probe and one because the registration of the vaginal probe was erroneous. The final sample comprised ten healthy women with an average age of 36.9 (8.19) years, a BMI of 20.76 (1.57) kg/m^2^, and a rating of four out of five on the Oxford Scale. Two women presented with constipation, and none had undergone gynecological surgery nor suffered from any other illness.

### 3.1. Isolated-Muscle Activation of the GM and PF in Static Tests and at Different Walking and Running Speeds

The peak-and-average-amplitude data are shown in Table 1 and Table 2, respectively. The muscle-activation values of the right GM during the static tests showed an average MVC of 149.43 (73.20) µV, which decreased to 50.16 (27.84) µV at a walking speed of 5 km/h. The race at speeds of 9 and 11 km/h yielded average amplitudes of 315% and 349% with respect to the %MVC. The values were similar in the contralateral muscle (*p* > 0.05).

The right PFM obtained a peak MVC of 101.26 (35.40) µV in the static tests (Table 1). It was activated at a walking speed of 5 km/h at 52%, and the values increased up to 76% at 7 km/h and 116% and 119% while running at 9 and 11 km/h, respectively. In the left PFM, the peak amplitude increased up to a value of 70% compared with the right at a speed of 5 km/h, later becoming equal to the data recorded in the contralateral muscle at speeds of 7, 9, and 11 km/h. It almost tripled during the race.

**Figure 2:**
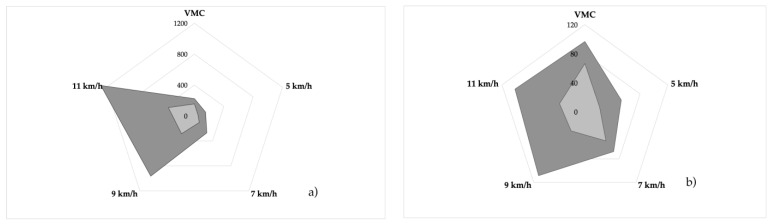
Representation of the contraction at the different speeds: (**a**) GM and (**b**) PFM. The graph represents the peak amplitude (dark grey) and average amplitude (light grey) in µV.

**Figure 3:**
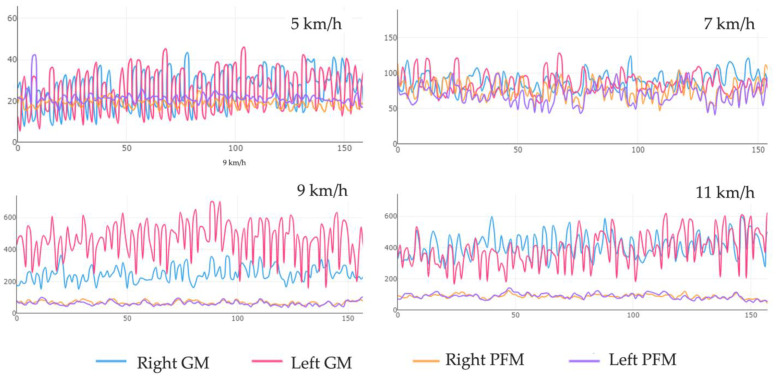
Electromyographic recordings at different speeds. The Y-axis represents the amplitude in µV; the X-axis represents the time in seconds. GM, gluteus medius; PFM, pelvic floor muscle.

The peak amplitude of the GM was reached while walking at 7 km/h (122.01%) and was exponential at 9 and 11 km/h (431.88% and 570.92%, respectively). It worked at half its average MVC while walking and almost tripled while running. However, the peak MVC of the PFMs was reached only while running at 9 and 11 km/h (115.5%) and did not exceed half of the average MVC while walking or running (Figure 2). The differences found in each of the tests carried out were statistically significant (*p* < 0.001) for both the GM and bilateral PFM (Figure 3).

### 3.2. Isolated-Muscle Ratios of the GM and PF in Static Tests and at Different Running Speeds

Physically active, nulliparous female runners without lower-limb dysmetria showed similar activation values in the GM and PFM to their respective contralateral muscles in all the tests (*p* > 0.05). The PFM only showed an unequal ratio between both sides at the 7 km/h pace.

**Figure 4:**
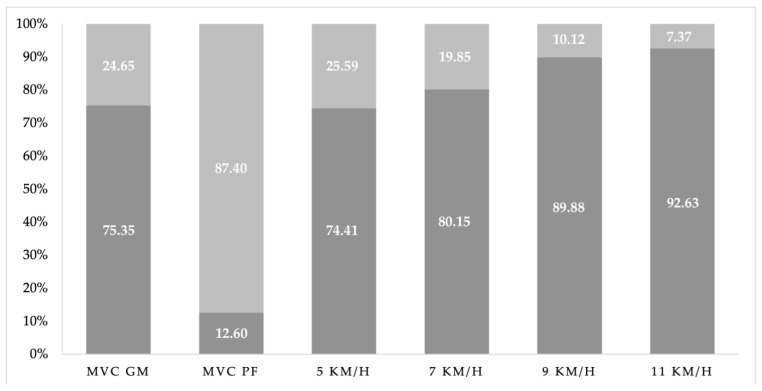
Representation of the global relationship while static and at different walking and running speeds. The graph represents the GM (dark grey) and PFM (light grey).

The average amplitude of the PFM was 72.99 µV (109.8% VMC) during the MVC of the GM. The average amplitude of the GM was 6.84 µV (4.57% MVC) during the MVC of the PFM. However, the global activity of the GM (75.3%) was predominant over the PFM (24.6%) while static and walking, with ratios of 3/1 and 4/1, respectively (Figure 3). The ratio reached 9/1 (GM/PFM) while running at 9 and 11 km/h. However, the PFM relationship was reversed (87.4%) compared with the GM in the static test (Figure 4). Despite the predominance of the GM over the PFM, no statistically significant relationships (*p* > 0.050) were found between them in any of the tests. However, significant direct associations were found in each muscle with its contralateral muscle (*p* < 0.05) in all tests.

## 4. Discussion

This study aimed to record the activity of the PFMs and GM muscles in healthy and physically active women using EMG during gait cycles at different speeds. The values without any voluntary contraction indicate PFM pre-activity and reflex activity while walking and running, with values varying between 37 and 63% of EMGmean and 59 and 119% of EMGpeak, similar to the findings of Luginbuehl et al. [35], Leitner et al. [35,43,44], and Williams [20] but different to those of Koening et al. [34]. The MVC test in a usual supine position [20] was chosen instead of in a standing position [35,43,44] because there is evidence that baseline PFM activity depends on the body position and is highest while standing [47,48]. While it is effective to normalize results as a percentage of the MVC to provide context to findings, caution is advised when interpreting the PFM-activation level (as a % of the MVC) achieved during gait [20]. 

The results of Backer et al. [49] and Kelly et al. [50] show an average GM activation at a speed of 10 km/h, which is below that shown in our results, with Backer et al. obtaining a value of 25% of their MVC and Kelly et al. obtaining 85.8% at the initial contact and 34.6% of the MVC during the rest of the race cycle. These results are similar to those obtained by Chen et al., in which an average activation of the GM of 97% of its MVC was obtained at an exhaustive speed, compared with our maximum of 11 km/h. Our results showed higher values of 345.3% and 392.8% of the MVC on the right side and similar values on the left side. In 2019, Foch et al. studied the activation of the GM in habitual runners of at least 10 km/week, without specifying the speed. They obtained values of approximately 88% of the average activation with respect to the MVC. These values are closer to those we calibrated at 7 km/h—89.98% for the right side and similar for the left side—and are above those we calculated at 5 km/h—52.2% and 39.3% for the right and left sides, respectively [51].

Kelly et al. showed an activation peak of 247.4 µV at a speed of 10 km/h in the GM, without specifying the side [50]. The results obtained in our study are quadruple those obtained by Kelly et al. at 9 km/h.

Our findings indicate that the maximal-voluntary-contraction percentage for the GM increased exponentially with the running speed, nearly tripling during the 11 km/h run. These results correlate with previous studies showing that the walk-to-run transition frequently occurs at approximately 7.0 km/h, and specific peak activations in the muscle synergies change before and after the walk-to-run transition [52]. Kibushi’s [53] findings in 2023 revealed that the peak amplitude of gastrocnemius muscle activity increases non-linearly with the walking speed. 

Kelly et al. [50] measured the activation of the vastus internus and vastus externus quadriceps muscles in a male and female group of regular runners at a speed of 10 km/h, obtaining peaks of 431.2 µV and 337.8 µV, respectively. Our results are more than double those of Kelly et al.’s on both sides at similar speeds. Backer et al. studied the behavior of other muscle groups while running, concluding that the gluteus maximus was activated at 5 km/h on average at approximately 20% of its MVC. The TFL was activated with an average amplitude of 7% of its MVC at that same speed after 3 min of running, which is below our results for the GM [49].

The human PFMs operate synergistically with a wide variety of muscles, which has been suggested to be an important contributor to continence and pelvic stability while performing functional tasks. The relationship between the PFMs and other hip-stabilizing muscles (e.g., the gluteus maximus [54] and external hip rotators [55]) has been proven electromyographically. The synergistic activation between the PFMs and the abdominal muscles is also supported by several authors [5,56], who have identified synergism during the contraction of the PFMs and the hip adductor and gluteal muscle contraction [5] in women with normal PFMs or those with PFM pathology. However, these findings should be interpreted with caution, given the studies included in this review did not use standardized methods to select participants, sample sizes, and EMG.

Overall, the coactivation of the PFMs [20,35,43,44] and GM [41,42,57] muscles plays a crucial role in maintaining stability, pelvic control, and proper lower-limb movement while walking and running. Our results indicate the activation of both throughout the gait cycle (Figure 3). As the walking or running speed increases, the coactivation of the PF and GM muscles increases correspondingly. This suggests that these muscles work together to provide the necessary stability and support at higher gait speeds. 

The PFMs did not show greater activation than the GM in the static or dynamic tests. However, the PFM activation exceeded its 100%aMVC during voluntary GM activation, while the GM activation reached an average of 4.5% during voluntary PFM activation. While people can contract their PFMs in isolation, it is generally impossible for people to activate their gluteal maximus muscles (GMMs) without coactivating their PFMs [58,59]. Junginger et al. [2] obtained similar results using functional magnetic resonance imaging, where the PFM activation reached an average of 26% of the MVC during voluntary GMM activation, although with lower values. Considering that the gluteal maximus and medius perform similar actions during gait, one would expect the PFMs to increase their activation, like the GM, according to speed, maintaining the same relationship between both. However, this study demonstrated that the GM/PFM ratio in women was 3/1 and 4/1 in the supine position and while walking. However, the ratio reached up to 9/1 while running. Therefore, the relationship is not directly proportional in phase while walking or running. Previous research has only explored PFM activation in response to isolated and voluntary gluteal contractions [1,2,5,58,59]. How this relationship may be altered during dynamic activities, such as gait, where different neural circuitry is involved, remains unknown. The PFMs are activated in response to other factors, such as modulations in IAP. Herein, we only tested our hypothesis that PFM and GM activation occur together while walking and running in nulliparous, physically active female runners throughout the gait cycle at different speeds.

The main limitations of this study could be attributed to, first, the small sample size, analyzing only females, and lack of a control group. Second, we did not consider how high- vs. low-impact activities or sports differentially affect these muscles [60]. Third, PF dysfunction (PFD) was not registered. Future investigations should consider using questionnaires and clinical or urodynamic tests to confirm the presence of PFD. However, no women reported leaking urine during the tests. Finally, we chose to use vaginal surface EMG in this study because the perianal recordings are likely to capture the responses primarily from the puborectalis and anal sphincters [61]. Some participants stated that the intravaginal probe, despite its light weight, caused a sensation of falling and discomfort and could pre-activate the SP, an issue that is not reflected in our results. Previous work has also shown no difference in the peak or mean root-mean-squared muscle activity of PFM EMG signals recorded vaginally or perianally [62]. Future research may expand on these findings by recording the responses of PFM activity and IAP to other forms of gait and during other dynamic tasks (e.g., jumping) [63] in a wider range of healthy individuals, other muscles, and women with PFD or differences between their dominant and non-dominant sides.

## 5. Conclusions

As the walking or running speed increases, the coactivation of the PF and GM muscles in nulliparous, physically active female runners increases correspondingly. This suggests that these muscles work together to provide the necessary stability and support at faster gait speeds. However, this study demonstrated that the GM/PFM ratio was 3/1 and 4/1 in the supine position or while walking, but the ratio reached 9/1 while running. Therefore, the relationship is not directly proportional in phase while walking or running. The GM triples its MVC at high speeds, while the PFM only reaches half of its maximum contraction.

## Figures and Tables

**Table 1:** Maximum muscle-activation (peak amplitude) values in static conditions and at different running speeds.

Muscle	MVC		5 km/h			7 km/h			9 km/h			11 km/h			*p*-Value *
	Mean (SD) ^a^	95% CI	Mean (SD) ^a^	95% CI	%MVC	Mean (SD) ^a^	95% CI	%MVC	Mean (SD) ^a^	95% CI	%MVC	Mean (SD) ^a^	95% CI	%MVC	
RGM	224.88 (118.92)	139.8–309.95	159.02 (70.83)	108.3–209.7	101.35 (95.38)	282.2 (90.48)	212.6–351.7	175.43 (150.63)	1069.06 (1036.32)	327.7–1810.4	796.85 (1289.96)	1199.94 (960.80)	512.6–1887.2	848.23 (1182.10)	<0.001
LGM	221.30 (126.49)	130.8–311.8	146.94 (52.75)	109.2–184.6	79.59 (48.77)	262.21 (102.81)	183.1–341.2	172.17 (110.77)	857.90 (763.90)	311.4–1404.3	538.43 (666.13)	1347.4 (1492.6)	279.6–2415.2	986.82 (1350.93)	<0.001
RPF	101.26 (35.40)	75.9–126.5	48.34 (29.59)	27.17–69.5	52.92 (39.96)	66.68 (53.93)	25.2–108.1	76.03 (71.79)	106.602 (71.42)	55.5–157.6	116.83 (85.70)	106.716 (43.12)	75.86–137.5	119.64 (66.60)	<0.001
LPF	92.17 (40.86)	62.9–121.4	56.88 (30.55)	35.0–78.7	70.04 (42.87)	68.14 (43.84)	34.4–101.8	76.02 (59.31)	110.29 (87.33)	47.8–172.7	115.09 (54.04)	95.96 (52.58)	58.3–133.5	112.71 (48.25)	0.137

RGM, right GM; LGM, left GM; RPFM, right pelvic floor; LPFM, left pelvic floor; MVC, maximum voluntary isometric contraction; km/h, kilometers per hour; SD, standard deviation; CI, confidence interval. Significance level was set at *p* < 0.05. ^a^ Microvolt (µV) measurement unit. (*) *p*-Value based on Friedman test.

**Table 2:** Muscle-activation values (average amplitude) in static conditions and at different running speeds.

Muscle	MVC		5 km/h			7 km/h			9 km/h			11 km/h			*p*-Value *
	Mean (SD) ^a^	95% CI	Mean (SD) ^a^	95% CI	%MVC	Mean (SD) ^a^	95% CI	%MVC	Mean (SD) ^a^	95% CI	%MVC	Mean (SD) ^a^	95% CI	%MVC	
RGM	149.43 (73.20)	97.1–201.8	50.16 (27.8)	30.2–70.0	52.12 (67.15)	96.48 (49.39)	58.5–134.4	89.98 (83.08)	315.081 (236.06)	146.2–483.9	345.3 (538.22)	349.21 (278.77)	149.7–548.6	392.48 (602.20)	<0.001
LGM	162.10 (83.91)	102.0–222.13	43.55 (30.9)	21.4–65.7	39.23 (53.32)	114.41 (107.94)	31.44–197.3	114.58 (163.79)	254.76 (197.71)	113.3–396.2	234.75 (322.90)	359.06 (359.76)	101.7–616.4	352.83 (515.71)	<0.001
RPF	68.92 (26.005)	50.3–87.5	22.06 (11.0)	14.2–29.9	36.99 (26.01)	26.63 (19.72)	11.4–41.7	46.21 (43.51)	31.33 (13.29)	21.8–40.8	52.19 (32.48)	36.69 (18.34)	23.2–49.8	61.44 (40.14)	<0.001
LPF	63.91 (29.30)	42.9–84.88	20.66 (8.50)	14.5–26.7	38.23 (22.35)	71.85 (35.09)	31.9–75.6	84.12 (13.72)	33.06 (15.20)	22.1–43.9	56.52 (22.34)	36.85 (18.97)	23.2–50.4	63.25 (26.43)	0.137

RGM, right GM; LGM, left GM; RPFM, right pelvic floor; LPFM, left pelvic floor; MVC, maximum voluntary isometric contraction; km/h, kilometers per hour; SD, standard deviation; CI, confidence interval. Significance level was set at *p* < 0.05. ^a^ Microvolt (µV) measurement unit. (*) *p*-value based on Friedman test.

## Data Availability

The data presented in this study are available upon request from the corresponding author. The data are not publicly available due to privacy and ethical restrictions.
